# Fluoroquinolone and Macrolide Treatment Failure in Pneumococcal Pneumonia and Selection of Multidrug-Resistant Isolates

**DOI:** 10.3201/eid0909.020810

**Published:** 2003-09

**Authors:** Emilio Pérez-Trallero, José M. Marimon, Luis Iglesias, Julián Larruskain

**Affiliations:** * Hospital Donostia, San Sebastián, Spain; † Universidad del País Vasco, San Sebastián, Spain

**Keywords:** treatment failure, drug resistance, *Streptococcus pneumoniae*, serotype 3, clarithromycin, ciprofloxacin, levofloxacin, quinupristin-dalfopristin, telithromycin, in vivo selection

## Abstract

*Streptococcus pneumoniae* serotype 3, isolated from a penicillin-allergic patient and initially susceptible to fluoroquinolones, macrolides, lincosamides, quinupristin-dalfopristin, and telithromycin, became resistant to all these drugs during treatment. Mutations in the *parC* and *gyrA* and in the 23S rRNA and the ribosomal protein L22 genes were detected in the resistant isolates.

Macrolide antimicrobial drugs and new fluoroquinolones have become good therapeutic choices in the treatment of penicillin-resistant *Streptococcus pneumoniae* infections and in penicillin-allergic patients with pneumococcal pneumonia. Until now, clinical failures of fluoroquinolones during treatment of pneumococcal infections have rarely been reported (*1*–*3*) and development of resistance in *S*. *pneumoniae* to quinupristin-dalfopristin and telithromycin during or after treatment with a macrolide or a combination of macrolide and quinolone antibiotics has never been reported.

We describe failure of treatment of pneumococcal pneumonia in a 71-year-old man, who was allergic to penicillin and had a history of chronic obstructive pulmonary disease. During treatment, isolates that were susceptible to levofloxacin, clarithromycin, clindamycin, quinupristin-dalfopristin, and telithromycin became resistant.

## The Study

*S*. *pneumoniae* were identified to the species level by their colony morphology, optochin sensitivity, and the bile solubility tests. Serotyping was performed by the Quellung reaction (Quellung antisera, Staten Seruminstitut, Copenhagen). MICs of the antibiotics and criteria of susceptibility and resistance, otherwise not indicated, were those of the broth microdilution procedure described by the National Committee for Clinical Laboratory Standards (NCCLS) (*4*). The agar dilution method (*5*) and the E-test, as referred to in Table 1, were performed to expand the range of dilutions available in the broth microdilution trays. No discordance was observed in the susceptibility results, which were obtained by using the broth microdilution, the agar dilution, or the elution E-test.

**Table 1:** In vitro antibiotic susceptibilities determined by agar dilution in *Streptococcus pneumoniae* isolates

Drug	MIC (μg/mL) for each isolate/specimen collected, by date				
	1st isolate/sputum, 1-26-2002	2nd isolate/sputum, 2-8-2002	3rd isolate/sputum, 2-20-2002	4th isolate/sputum, 2-20-2002	5th isolate/pleural fluid, 02-26-2002
Ciprofloxacin	2	32	16	16	16
Levofloxacin	2	16	16	16	16
Moxifloxacin	1	4	4	4	4
Gemifloxacin	0.06	0.5	0.5	0.5	0.5
Erythromycin (14-m **macrolide**)	<0.25	2	>128	>128	>128
Clarithromycin (14-m **macrolide**)	<0.25	2	>128	>128	>128
Azithromycin (15-m **macrolide**)	0.5	4	>128	>128	>128
Midekamycin (16-m **macrolide**)	<0.5	4	128	64	16
Clindamycin	<0.25	≤0.25	16	16	16
Quinupristin-dalfopristin ^a^	1	32	>32	>32	2
Telithromycin	≤0.12	2	8	16	0.5

^a^ Determined by E-test; m, membered.

Molecular typing methods (pulsed-field gel electrophoresis [PFGE], BOX-polymerase chain reaction [PCR], and multilocus sequence typing) of the isolates were performed according to previously described protocols (*6*). Presence of the *mefA,* *ermB,* and *ermA* (*ermTR*) genes and point mutations at Ser-79 in the *parC* and at Ser-81 in the *gyrA* genes were detected as previously described (*6*). Fragments of the domains II and V of the 23S rRNA genes and of the genes encoding ribosomal proteins L4 and L22 were amplified by using the primers and conditions previously described (*7*,*8*). Amplification products were sequenced after purification.

## Case Description

In January 2002, a 71-year-old man, who was allergic to penicillin and had a history of chronic obstructive pulmonary disease, was hospitalized due to pneumonia. The first *S*. *pneumoniae* strain was isolated from sputum obtained before antibiotic treatment with intravenous levofloxacin (500 mg once a day for 13 days) was begun. On day 4, intravenous clarithromycin (500 mg twice a day) was added but withdrawn after 4 doses. On day 14, clinical and radiologic conditions had deteriorated, and treatment was changed to intravenous clarithromycin (500 mg) and intravenous ciprofloxacin (200 mg) twice a day for 7 days. On the same day, a second pneumococcal isolate resistant to levofloxacin and clarithromycin but susceptible to clindamycin was obtained (Table 1). The MIC of clarithromycin for this second isolate was 2 μg/mL by the double-disk test (*9*) and showed that the susceptibility of clindamycin was not modified after the erythromycin induction. Initially, this second isolate was incorrectly reported as clarithromycin susceptible because of an erroneous record of the result of the disk-diffusion method. On day 24, the patient was discharged with oral clarithromycin. Twenty-four hours later, the patient was readmitted with exacerbation of the respiratory infection and cor pulmonale, and two pneumococcal isolates resistant to levofloxacin, clarithromycin, and clindamycin were found within 6 hours. The patient received trimethoprim-sulfamethoxazole for 5 days; a fifth pneumococcal isolate was found from a pleural effusion specimen. The pneumonia completely resolved after 10 days of treatment with vancomycin. The five *S*. *pneumoniae* serotype 3 isolates recovered over a 32-day period had the same PFGE, BOX-PCR patterns, and multilocus sequence typing (ST180) results.

All *S. pneumoniae* isolates were susceptible to penicillin (MIC <0.03 μg/mL), trimethoprim-sulfamethoxazole (MIC <0.5/9.5 μg/mL), tetracycline (MIC <2 μg/mL), chloramphenicol (MIC <2 μg/mL), and vancomycin (MIC=0.5 μg/mL). The first isolate was susceptible to both macrolides and fluoroquinolones. This isolate had a levofloxacin MIC of 2 μg/mL, confirmed by all susceptibility methods used (E-test, broth microdilution, and agar dilution), although it had a point mutation in the *gyrA* gene, as shown in Table 2.

**Table 2:** Point mutations in fluoroquinolone targets and macrolide-lincosamide-streptogramin-resistance determinants and ribosomal mutations in *Streptococcus pneumoniae* isolates

Location	1st isolate	2nd isolate	3rd isolate	4th isolate	5th isolate
*parC*	-	Ser-79	Ser-79	Ser-79	Ser-79
*gyrA*	Ser-81	Ser-81	Ser-81	Ser-81	Ser-81
*ermA, ermB* and *mefA* genes	-	-	-	-	-
Ribosomal protein L4	-	-	-	-	-
Amino acids insert in ribosomal protein L22	-	RTAHIT^a^	RTAHIT	RTAHIT	-
23S rRNA gene (domain II)	-	-	-	-	-
23S rRNA gene (domain V)	-	-	A2058G	A2058G	A2058G

^a^Sequence data GenBank AY140892.

For the second isolate, MICs of macrolides, quinupristin-dalfopristin, and telithromycin were higher than those for the first isolate, and a 18-base insert in the sequence of the gene encoding the ribosomal protein L22 was detected. The result, deduced from the corresponding ribosomal protein, was a six–amino acid (RTAHIT) insertion between amino acids T108 and V109 (GenBank accession no. AY140892). The third and fourth isolates, with resistance to macrolides, clindamycin, quinupristin-dalfopristin, and the highest telithromycin MICs of all the isolates, had an A2058G (*Escherichia coli* numbering) mutation in the sequence of the gene corresponding to the domain V of the 23S rRNA as well as the 6–amino acid insert in the ribosomal protein L22. The four alleles encoding the 23S rRNA gene had the A2058G mutation. The sequences of the fifth isolate, resistant to macrolide antibiotics and clindamycin, with an intermediate susceptibility to quinupristin-dalfopristin, indicated a mutation at position 2058 of domain V, but no insert was found in the ribosomal protein L22.

## Conclusions

Since the introduction of antimicrobial drugs in therapy, *S. pneumoniae* has shown a strong ability to acquire resistance to the progressive introduction of new antibiotics to treat it.

Surveillance studies suggest that the levels of resistance to macrolide antibiotics in *S. pneumoniae* are high and are still rising (*9*,*10*). Ketolides, of which telithromycin is the first to be registered for clinical use, and quinupristin-dalfopristin are new compounds belonging to the macrolide-lincosamide-streptogramin B (MLSb) class of antimicrobial agents. One of the main advantages attributed to these two new families of antibiotics is their ability to retain activity against most resistant pneumococcal isolates (*11*). Recently, mutations in the 23S rRNA genes and in ribosomal proteins L4 and L22 have been identified in macrolide-resistant *S. pneumoniae,* although the predominant mechanisms of resistance are mediated by *ermB* or by *mefA* genes (*12*). The combination of the mutation in the domain V of the 23S rRNA and the insertion in the L22 ribosomal protein gene has not been previously described in *S. pneumoniae* isolated in vivo or in vitro. In these strains, a high level of resistance to **14**-, **15**-, and 16-membered ring **macrolide**s and to clindamycin and resistance to quinupristin-dalfopristin and telithromycin were observed. The continued use of clarithromycin in the presence of an isolate with an insertion in the L22 ribosomal protein gene may have led to the selection of the isolates with the double mutations, L22 and 23S rRNA genes, associated with combined resistance to telithromycin and quinupristin-dalfopristin, although neither of these antibiotics was used. The A2058G mutation in all four 23S rRNA genes alone slightly increased both quinupristin-dalfopristin and telithromycin MICs as seen in the fifth isolate. The L22 insertion alone, as observed in the second isolate, was enough to confer a high level of quinupristin-dalfopristin resistance and also increased the telithromycin MIC to 2 μg/mL. The combination of both mutations (L22 insertion and A2058G mutation in the 23S rRNA genes) led to high level of resistance to telithromycin and increased the quinupristin-dalfopristin MIC (third and fourth isolates).

The first isolate was susceptible to all antibiotics tested, and although it had a point mutation in the *gyrA* gene, it had no phenotypic expression. In mutants obtained in vitro, other authors observed point mutation in the *gyrA* gene without mutation in the *parC* gene with or without phenotypic expression of quinolone resistance (*13*). Nevertheless, using fluoroquinolones to treat a strain that had an existing, but unapparent, first-step mutation in the *gyrA* gene, probably favored the development of the high level of resistance to fluoroquinolones observed in the later isolates. Fluoroquinolone resistance in clinical isolates of *S. pneumoniae* is still infrequent, but in some places, the resistance has been increasing (*9*,*14*–*16*).

Until now, most erythromycin- or fluoroquinolone-resistant pneumococci had belonged to only a few serotypes. Finding an erythromycin-resistant serotype 3 was unusual, and the isolation of a fluoroquinolone-resistant serotype 3 *S. pneumoniae* was the exception, if ever reported. Penicillin or another appropriate β-lactam antibiotic could have been a valid therapeutic option in the absence of allergy to penicillin. Serotype 3 is considered the most virulent of *S. pneumoniae* serotypes*,* and it is commonly associated with invasive disease in adults. Most serotype 3 isolates have broad antibiotic susceptibility (*17*). A fatal infection associated with a multiply drug-resistant *S. pneumoniae* serotype 3 was first reported in 1988 (*18*). This strain was resistant to erythromycin, clindamycin, and tetracycline.

The therapeutic failure and selection of resistance to several antibiotics by *S. pneumoniae*, the emergence of new mechanisms of resistance to macrolides in clinical isolates of *S*. *pneumoniae,* and the appearance of multidrug resistance in a serotype 3 isolate (ST180) evoke concern.
